# The Utility of Comprehensive Metabolic Panel Tests for the Prediction of Bronchopulmonary Dysplasia in Extremely Premature Infants

**DOI:** 10.1155/2019/5681954

**Published:** 2019-10-20

**Authors:** Xueyu Chen, Binchun Lin, Xiaoyun Xiong, Panpan Sun, Yanqing Kong, Chuanzhong Yang

**Affiliations:** ^1^Department of Neonatology, Affiliated Shenzhen Maternity & Child Healthcare Hospital, Southern Medical University, Hongli Rd, 2004, 518507 Shenzhen, China; ^2^Department of Pathology, Affiliated Shenzhen Maternity & Child Healthcare Hospital, Southern Medical University, Hongli Rd, 2004, 518507 Shenzhen, China

## Abstract

**Background:**

Comprehensive metabolic panel tests (CMP) are routinely performed in extremely premature infants within the first days of life. The association between the parameters of first postnatal CMP and the risk of bronchopulmonary dysplasia (BPD) remains elusive.

**Methods:**

A retrospective analysis was performed to evaluate the correlation between the parameters of first postnatal CMP and the risk of BPD in a cohort of extremely premature infants (born with a gestational age less than 28 weeks or a birth weight less than 1000 grams) at the neonatal intensive care unit, Shenzhen Maternity and Child Healthcare Hospital, from January 2016 to October 2018. A multivariant regression model was built to assess the association of the first postnatal CMP with the development of BPD.

**Results:**

A total of 256 extremely premature infants were included in this study. BPD developed in 76 (29.7%) infants. The first CMP in these infants was performed at 5 to 8 days after birth. The levels of blood urea nitrogen (BUN) and magnesium were significantly higher in infants with BPD compared to infants with no BPD (10.2 versus 7.5 mmol/L, *P* < 0.001 and 0.9 versus 0.8 U/L, *P* = 0.001, respectively) whereas the level of alkaline phosphatase (ALP) and total protein was significantly lower in infants with BPD (215.5 versus 310.0 U/L, *P* = 0.002 and 41.2 versus 42.9 g/L, *P* = 0.037, respectively). Multiple analysis showed that a higher level of BUN (>8.18 mmol/L) was independently associated with BPD (OR 3.261, 95% CI 1.779-5.978).

**Conclusion:**

Our findings indicate that a higher postnatal BUN level (>8.18 mmol/L) may be a predictor for the development of BPD in extremely premature infants.

## 1. Introduction

Bronchopulmonary dysplasia (BPD) occurs in 40% of extremely preterm birth, with pathological characters of arrested alveolar and vascular development [1, 2]. BPD severely compromises the short- and long-term well-being of extremely premature infants by increasing the risk of respiratory infection, asthma, and chronic obstructive pulmonary distress (COPD) in their later life [3–5].

Despite the known risk factors for BPD, little improvement has been made in reducing the prevalence of BPD. Therefore, studies identifying the vulnerable population and the crucial prophylactic window are of paramount importance. Antenatal and postnatal factors have been related to the deterioration of the lung development, including gestational age at birth, maternal complications, placenta abnormalities, infection, and persistent mechanistic ventilation [6, 7]. Recently, nutrition status was found associating with the development of BPD [8]. Alteration of lipid metabolism was reported in BPD or hyperoxia-induced injury [9, 10]. However, a complicated test for lipid metabolites may compromise its application in daily practice. This study is aimed at exploring the association between the parameters in a comprehensive metabolic panel (CMP) and the development of BPD in a cohort of extremely premature infants. We hypothesized that metabolism status during early postnatal life may be useful to discriminate the high-risk infants of BPD.

## 2. Materials and Methods

### 2.1. Study Population

This is a retrospective cohort study. All extremely premature infants admitted to the Neonatal Intensive Care Unit (NICU), Shenzhen Maternity and Child Healthcare Hospital, from January 2016 to October 2018 were included in this study. We excluded infants withdrawn from intensive care before attempting extubation. Infants referred from other hospitals were also excluded because their first CMP parameters were missed. Infants with congenital abnormalities were excluded as well.

### 2.2. Definition of Clinical Variables

Extreme prematurity was defined as birth at a gestational age less than 28 weeks or birth weight less than 1000 grams. BPD was diagnosed when supplemental oxygen was required at 36 weeks postmenstrual age (PMA) or at discharge [11, 12]. Gestational diabetes mellitus (GDM) was defined according to blood glucose level with a 75 g oral glucose tolerance test (OGTT): fasting ≥ 5.3 mmol/L, 1 hour ≥ 10.6 mmol/L, or 2 hours ≥ 9.0 mmol/L [13]. Gestational hypertension was defined as a systolic blood pressure of ≥140 mmHg or diastolic blood pressure of ≥90 mmHg after 20 weeks of gestation [14]. Neonatal respiratory distress syndrome (NRDS) was diagnosed according to the clinical symptoms and chest X-ray. (Suspected) early-onset neonatal sepsis occurring within the first 72 hours of life was defined as the following criteria: a positive culture of blood and/or the presence of clinical signs of infection with abnormal chest radiograph profiles, hematological features, and maternal risk factors [15]. Patent ductus arteriosus (PDA) was diagnosed when the ratio of the left arterial to aortic root dimensions is ≥1.5 : 1, the ductal diameter is ≥1.5 mm, and the reversal of the diastolic flow in the descending aorta is demonstrated by echo [16]. Intraventricular hemorrhage (IVH) was diagnosed according to a cranial ultrasound and graded from I to IV. Antenatal steroid treatment was recorded if at least one dose of dexamethasone was administrated 12 hours before delivery. Surfactant treatment was considered if at least one course of surfactant was administrated.

### 2.3. Data Collection

Infants' clinical data were retrieved from the electronic medical record. The first postnatal CMP was performed within 5-8 days after birth. All CMP test was performed on UniCel DxC 800 Synchron (Beckman Coulter, Georgia) using the blood from the umbilical artery catheter of the infants.

### 2.4. Statistics

The sample size calculation was based on the BUN level from our clinical laboratory. At 90% power and *α* = 0.05, 53 infants in each group would be sufficient to detect a significant difference (PASS, version 11, NCSS, LLC, Utah). CMP parameters were displayed as median [interquartile range (IQR)] and analyzed by the unpaired *t*-test or nonparametric test, as appropriate. Categorical variables were described with numbers and percentages and analyzed by chi-square or Fisher's exact test accordingly. Multivariate and ordinal logistic regression was applied to identify the independent risk factors of BPD. The odds ratios (ORs) and 95% confidence interval (CI) were determined in logistic regression analysis. Subsequently, the receiver-operator curve (ROC) was adopted to calculate the cutoff values to dichotomize the continuous variables independently associated with the occurrence of BPD. Statistical analyses were performed using SPSS version 24 (IBM Corporation, NY).

The study was conducted in accordance with the Declaration of Helsinki and approved by the Shenzhen Maternity and Child Healthcare Hospital Institutional Ethical Committee (No. [2019]-119).

## 3. Results

**Figure 1:**
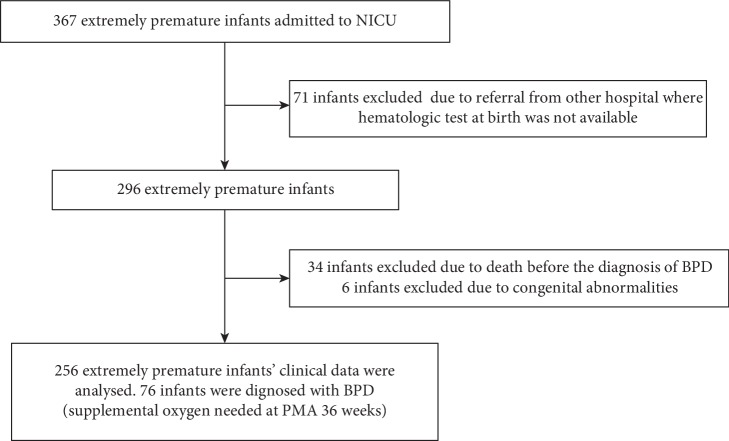
Flowchart of case selection.

A total of 367 extremely premature infants were admitted to our NICU during the study period. Seventy-one infants referred from other hospitals were excluded because their first CMP results were missed. Thirty-four infants were excluded due to withdrawal from the intensive care prior to attempting extubation. Six infants with congenital abnormalities were excluded as well. As a result, 256 infants were included in our analysis. The diagnosis of BPD (oxygen needed at 36 wk, PMA, or discharge) was made in 76 (29.7%) infants (Figure 1). 228 (89.0%) infants were born before 28 weeks and 28 (10.9%) infants were born after 28 weeks with a birth weight lower than 1000 grams. The median of GA at birth was 26.9 (IQR: 25.7-27.4) weeks. The clinical characteristics are described in Table 1.

### 3.1. Clinical Characteristics of the Cohort

BPD infants have lower gestational age (25.9 versus 27.1 weeks, *P* < 0.001), birth weight (788 versus 915 grams, *P* < 0.001), 1-minute Apgar score (5 versus 8, *P* = 0.001), 5-minute Apgar score (10 versus 10, *P* = 0.016), and lower rate of cesarean section delivery (17.1% versus 30.6%, *P* = 0.019), compared to infants with no BPD. Furthermore, BPD infants have higher rate of surfactant treatment (90.8% versus 64.8%, *P* = 0.019), intubation (84.2% versus 48.9%, *P* < 0.001), (suspected) early-onset neonatal sepsis (47.4% versus 19.4%, *P* < 0.001), PDA (57.9% versus 31.7%, *P* < 0.001), and grades III and IV IVH (14.5% versus 5.0%, *P* = 0.012, Table 1).

### 3.2. Comparison of CMP Parameters by BPD Status

The comparison of first CMP parameters at postnatal days 5-8 between infants with and without BPD is summarized in Table 2. The blood urea nitrogen (BUN) and Mg were significantly higher in infants with BPD compared with no BPD infants (10.2 versus 7.5 mmol/L, *P* < 0.001; 0.9 versus 0.8 mmol/L, *P* = 0.001, respectively). The levels of alkaline phosphatase (ALP) and total protein were significantly lower in infants with BPD compared with those in no BPD infants (215.5 versus 310.0 U/L, *P* = 0.002, and 41.2 versus 42.9 g/L, *P* = 0.037, Table 2).

### 3.3. Identifying the Independent Risk Factors for BPD

These potential confounders were subsequently entered to the multivariable regression model. We found that the risk of BPD was independently associated with birth weight (OR: 0.996, 95% CI: 0.994-0.999, *P* = 0.006), intubation (OR: 3.521, 95% CI: 1.249-9.925, *P* = 0.017), (suspected) early-onset neonatal sepsis (OR: 6.200, 95% CI: 2.639-14.568, *P* < 0.001), PDA (OR: 2.527, 95% CI: 1.127-5.664, *P* = 0.024), ALP (OR: 0.996, 95% CI: 0.993-1.000, *P* = 0.022), and BUN (OR: 1.125, 95% CI: 1.007-1.256, *P* = 0.037, Table 3) level detected at postnatal days 5-8.

### 3.4. Calculation of the Cutoff Value for BUN and ALP and Validation

The receiver-operator curve was used to calculate the cutoff value of the BUN and ALP level measured at postnatal days 5-8 for optimally assessing the risk of BPD ([Supplementary-material supplementary-material-1]). A BUN level of 8.18 mmol/L was concluded as the best cutoff value with the area under the curve (AUC: 0.680), sensitivity (0.714), specificity (0.566), and Youden's index (0.280, Table 4). Since AUC for ALP was lower than 0.5 in the ROC, further analysis for ALP was not performed. The clinical outcome of this cohort was laminated by the BUN level (Table 5). Besides the effect on the development of BPD (42.0% versus 18.2%, *P* < 0.001), a higher level of BUN (>8.18 mmol/L) increased the risk for PAH, ROP requiring interventions, severe IVH, and the duration of intubation compared with infants with lower BUN level (<8.18 mmol/L) [10.9% versus 2.7%, *P* = 0.024; 16.8% versus 5.5%, *P* = 0.012; 12.6% versus 2.7%, *P* = 0.012; and 1.9 versus 0.4 day, *P* = 0.002, Table 5]. Furthermore, ordinal logistic regression was performed to examine the contribution of higher BUN levels to the severity of BPD. Compared to infants with BUN < 8.18 mmol/L, infants with BUN > 8.18 mmol/L showed a 1.5-fold risk for moderate-to-severe BPD (Supplemental [Supplementary-material supplementary-material-1]).

## 4. Discussion

In this cohort of extremely premature infants, we found that several parameters of the first postnatal CMP were different between extremely premature infants with and without BPD. Further analysis indicates that a higher level of BUN (>8.18 mmol/L) was independently associated with the risk of BPD.

In current studies, we found gestational age, birth weight, 1- and 5-minute Apgar scores were protective factors of BPD, while C-section, intubation, and PDA were risk factors of BPD, as demonstrated by numerous studies [7]. Additionally, suspected early-onset sepsis was found to be associated with a higher risk of BPD, which was confirmed by Ballard et al. [17]. Recently, Pan et al. found inflammatory cytokines and inflammasome activation, and further inhibiting surfactant expression, might be the underlying mechanism explaining the influence of intrauterine infection on lung development [18]. Besides, we found a higher proportion of surfactant treatment in BPD infants, which may be explained by severe fundamental lung condition in infants who developed BPD later.

Timely and accurate evaluation of the risk of BPD in extremely premature infants is pivotal for implementing prevention strategies. Efforts are made to determine the risk factors predisposing extremely premature infants to the development of BPD. Recently, La Frano et al. found an altered lipid metabolism in the umbilical cord blood from infants progressing to BPD later [10], highlighting the role of metabolism in postnatal lung development raised by Surate et al. in their elegant review [19]. In the present study, we found that the postnatal BUN level (>8.18 mmol/L) was an independent risk factor for BPD. Postnatal BUN is influenced by several factors, such as protein intake, liquid intake, hypotension, and dehydration. Infants with hypotension suffered from an increased risk of BPD [20]. In contrast, Bell et al. summarized the data from 5 RCTs in preterm infants showing that the restriction of water intake tends to decrease the risk of BPD [21]. Moreover, Malikiwi et al. reported that a lower daily caloric intake during the first 4 weeks of life contributes to the occurrence of BPD [22]. These conflicting findings reveal the pivotal role of nutrition management in BPD and highlight the need for further studies on this topic.

The association between a high level of BUN and an increased risk of BPD could be explained by experimental evidence. Arginine, the substrate of urea, is also a precursor of nitric oxide (NO), the very important small molecule playing a crucial role in neonatal pulmonary disease, like BPD and pulmonary hypertension (PAH). Zheng and his colleagues have reported a disruption in the urea cycle in PAH animals induced by monocrotaline [23]. Despite the difference between adult PAH and BPD-associated PAH [24], there are still similarities in those two models, such as response to NO [25]. Increased levels of BUN may be associated with less arginine converting to NO, therefore eliminating the beneficial role of NO in lung development and pulmonary arterial pressure. This speculation is partially supported by research showing that arginase inhibition suppresses angiogenesis *in vitro* [26] and significantly augmented the risk of developing PAH (3.430-fold) in infants with a higher level of BUN in this study.

We also found that the ALP level was significantly lower in infants with BPD compared to those with no BPD. Elevated ALP is a reliable marker of vitamin D (VD) deficiency. Supplementation of VD alleviates the hyperoxia-induced lung injury in newborn rats by stimulating alveolarization via lipopolysaccharide- (LPS-)toll like receptor 4 (TLR4) pathway and suppression of inflammatory cytokine interferon *γ* (INF*γ*) [27, 28]. Çetinkaya et al. reported an association between 25-OHD [29] and BPD. However, this association was not substantiated in other studies [30]. We identified a minor protective effect of ALP on BPD in the current study (OR: 0.996), indicating that further studies are needed to clarify the association of ALP with BPD.

The main advantage of our research is the clinical applicability. CMP test is routinely performed in daily practice. Since BPD remains a challenge for neonatologists, identification of high-risk infants and timely intervention are pivotal for the prevention of BPD. However, our result should be interpreted with caution. Apart from the retrospective design, excluding the infants who had their intensive care withdrawn before the diagnosis of BPD leads to an inclusion bias in our study, since most of them are potential BPD candidates. Moreover, it would be interesting to explore the association between elevated BUN and BPD in PAH infants. We did not perform the analysis due to limited sample size (only 16 infants developed PAH in the current cohort). Furthermore, it would be interesting to investigate the dynamic change of BUN and ALP in the first month of life and its predictive value for BPD.

## 5. Conclusion

In conclusion, analysis of this extremely premature cohort indicated a high BUN level (>8.18 mmol/L) measured at postnatal days 5-8 which is independently associated with an elevated risk for BPD. This finding highlighted the relation between neonatal metabolism and the occurrence of BPD. Further experimental studies are needed to investigate the mechanism of nitrogen metabolism and its effect on lung development.

## Figures and Tables

**Table 1:** Maternal and neonatal characteristics of 256 extremely premature infants by BPD status.

Variables	Control (*N* = 180)	BPD (*N* = 76)	*Z*/*t*/*χ*^2^	*P* value
Gestational age [wk, M (Q1, Q3)]	27.1 (26.1, 27.6)	25.9 (24.5, 26.9)	-5.391	*P* < 0.001
Birth weight [gr, M (Q1, Q3)]	915 (816, 1037)	788 (687, 921)	-4.899	*P* < 0.001
Sex (male)	96 (53.3%)	48 (63.2%)	2.096	0.148
Gestational diabetes mellitus (GDM)	20 (11.1%)	6 (7.9%)	0.713	0.399
Gestational hypertension (GH)	17 (9.4%)	5 (6.6%)	0.651	0.420
Antenatal steroid	132 (73.3%)	60 (80.0%)	0.508	0.476
Delivery (C-section)	55 (30.6%)	13 (17.1%)	5.504	0.019
1-minute Apgar score [score, M (Q1, Q3)]	8 (5, 9)	5 (5, 8)	-3.192	0.001
5-minute Apgar score [score, M (Q1, Q3)]	10 (9, 10)	10 (8,10)	-2.415	0.016
Surfactant	127 (64.8%)	69 (90.8%)	5.468	0.019
Intubation	88 (48.9%)	64 (84.2%)	25.109	*P* < 0.001
(Suspected) early-onset sepsis	35 (19.4%)	36 (47.4%)	19.566	*P* < 0.001
Patent ductus arteriosus (PDA)	57 (31.7%)	44 (57.9%)	14.130	*P* < 0.001
Intraventricular hemorrhage (IVH, grades III and IV)	9 (5.0%)	11 (14.5%)	6.291	0.012

Data were displayed as median (interquartile range) or number (percentage). Wk: week; gr: gram; yr: year.

**Table 2:** Postnatal comprehensive metabolic panel (CMP) characteristics of 221 extremely premature infants by BPD status.

	Control (*N* = 180)	BPD (*N* = 76)	*Z*	*P* value
Total protein (g/L)	42.9 (39.2, 45.7)	41.2 (37.6, 43.8)	-2.083	0.037
Albumin (ALB, g/L)	24.8 (22.7, 27.1)	24.1 (21.8, 26.2)	-1.730	0.084
Alanine aminotransferase (ALT, U/L)	7.0 (6.0, 8.0)	6.0 (5.0, 8.0)	-1.266	0.205
Aspartate aminotransferase (AST, U/L)	25.0 (19.0, 32.0)	25.5 (19.8, 34.0)	-0.770	0.441
Alkaline phosphatase (ALP, U/L)	310.0 (212.5, 398.2)	215.5 (173.5, 330.7)	-3.074	0.002
Blood urea nitrogen (BUN, mmol/L)	7.5 (4.7, 10.7)	10.2 (7.5, 13.0)	-4.335	*P* < 0.001
Total calcium (Tca, mmol/L)	2.3 (2.2, 2.5)	2.3 (2.2, 2.5)	-0.374	0.709
Sodium (Na, mmol/L)	134.7 (131.3, 138.1)	134.2 (129.8, 137.6)	-1.378	0.168
Potassium (K, mmol/L)	4.6 (4.2, 4.9)	4.7 (4.3, 5.28)	-1.825	0.068
Magnesium (Mg, mmol/L)	0.8 (0.7, 0.9)	0.9 (0.8, 1.0)	-3.208	0.001
Creatine kinase-MB (CK-MB, U/L)	10.0 (7.0, 13.7)	10.0 (6.0, 15.0)	-0.559	0.576

Data were displayed as median (interquartile range). The CMP test was performed between postnatal days 5 and 8.

**Table 3:** Multivariate logistic regression of independent risk factors of BPD.

Variates	*β*	S.E.	Wald	*P*	OR (95% CI)
Birth weight (gr)	**-0.004**	**0.00**	**7.461**	**0.006**	**0.996 (0.994, 0.999)**
Gestational age (wk)	-0.296	0.202	2.149	0.143	0.744 (0.501, 1.105)
Intubation	**1.259**	**0.529**	**5.666**	**0.017**	**3.521 (1.249, 9.925)**
sEOS	**1.825**	**0.436**	**17.521**	**<0.001**	**6.200 (2.639, 14.568)**
C-section	0.006	0.552	0.000	0.991	1.006 (0.341, 2.972)
1-minute Apgar	-0.097	0.108	0.806	0.369	0.907 (0.734, 1.122)
5-minute Apgar	0.349	0.042	0.856	0.355	1.039 (0.958, 1.128)
PDA	**0.927**	**0.412**	**5.065**	**0.024**	**2.527 (1.127, 5.664)**
IVH grades III and IV	-0.406	0.717	0.320	0.572	0.667 (0.163, 2.719)
Surfactant	0.213	0.692	0.095	0.758	1.238 (0.319, 4.808)
Total protein (g/L)	0.039	0.042	0.856	0.355	1.039 (0.958, 1.128)
ALP (U/L)	**-0.004**	**0.002**	**5.209**	**0.022**	**0.996 (0.993, 1.000)**
Mg (mmol/L)	0.012	0.013	0.773	0.379	1.012 (0.986, 1.039)
Blood urea nitrogen (BUN, mmol/L)	**0.117**	**0.056**	**4.345**	**0.037**	**1.125 (1.007, 1.256)**

Abbreviations: gr: gram; wk: week; PDA: patent ductus arteriosus; IVH: intraventricular hemorrhage. sEOS: (suspected) early-onset sepsis is clinical or culture-proven sepsis diagnosed within 72 hours after birth.

**Table 4:** Calculation of cutoff value of BUN and ALP for discriminating BPD status.

Variable	AUC	Sensitivity	Specificity	Youden's index	Cutoff value
BUN (mmol/L)	0.680	0.714	0.566	0.280	8.18
ALP (U/L)	0.372	—	—	—	—

**Table 5:** Effect of BUN on BPD and other neonatal morbidities.

Morbidities	BUN < 8.18 mmol/L (110)	BUN > 8.18 mmol/L (119)	*β*/Z	*P* value	OR (95% CI)
BPD	20 (18.2%)	50 (42.0%)	1.182	*P* < 0.001	3.261 (1.779, 5.978)
PAH	3 (2.7%)	13 (10.9%)	1.488	0.024	4.430 (1.221, 16.069)
Intervened ROP	6 (5.5%)	20 (16.8%)	5.952	0.012	3.468 (1.319, 9.118)
IVH (grade III or IV)	3 (2.7%)	15 (12.6%)	3.535	0.012	5.049 (1.419, 17.962)
Intubated days	0.4 (0.0, 3.0)	1.9 (0.0, 18.0)	-3.155	0.002	—
nCPAP days	16.0 (7.5, 28.5)	21.2 (8.2, 32.0)	-1.191	0.234	—
Hospital stays	76.0 (65.0, 95.7)	79.0 (67.5, 109.0)	-1.672	0.094	—

Data were displayed as median (interquartile range) or number (percentage). BPD: bronchopulmonary dysplasia; PAH: pulmonary hypertension; ROP: retinopathy of prematurity; IVH: intraventricular hemorrhage; nCPAP: nasal continuous positive airway pressure.

## Data Availability

The data used to support the findings of this study are available from the corresponding author upon request.

## Abbreviations

BPD: Bronchopulmonary dysplasia
NICU: Neonatal intensive care unit
CMP: Complex metabolic panel
BUN: Blood urea nitrogen
ALP: Alkaline phosphatase
PMA: Postmenstrual age
GDM: Gestational diabetes mellitus
GH: Gestational hypertension
NRDS: Neonatal respiratory distress syndrome
PDA: Patent ductus arteriosus
IVH: Intraventricular hemorrhage
GA: Gestational age
BW: Birth weight
ROC: Receiver-operator curve
OR: Odds ratio
95% CI: 95% confidence interval
ROP: Retinopathy of prematurity
PAH: Pulmonary hypertension
nCPAP: Nasal continuous positive airway pressure.

## Supplementary Materials

Supplementary Materials Supplemental Figure 1: ROC for BUN and ALP. Supplemental Table 1: ordinal logistic regression of BPD status defined by NIH.
