# Supplementary material for: The Utility of Comprehensive Metabolic Panel Tests for the Prediction of Bronchopulmonary Dysplasia in Extremely Premature Infants
Source: Dis Markers. 2019 Oct 20;2019:5681954. doi: 10.1155/2019/5681954 (PMC6854975; doi:10.1155/2019/5681954)
Supplement: Supplementary Materials — Supplemental Figure 1: ROC for BUN and ALP. Supplemental Table 1: ordinal logistic regression of BPD status defined by NIH. [file 5681954.f1.docx]

Supplemental Figure1 ROC for BUN and ALP

Supplemental Table1 Ordinal logistic regression of BPD status defined by NIH

| Variates | | OR | Log Odds | P | 95% C.I. |
| --- | --- | --- | --- | --- | --- |
| Threshold | No BPD^1^ | 0.166 | -1.798 | 0.020 | (-3.314, -0.282) |
|  | Mild BPD^2^ | 3.551 | 1.267 | 0.101 | (-0.246, 2.781) |
|  | Moderate BPD^3^ | 20.114 | 3.000 | <0.001 | (1.439, 4.563) |
| Birth Weight (gr.) | | 0.999 | -0.001 | 0.046 | (-0.003, 0.000) |
| ALP (U/L) | | 0.999 | -0.001 | 0.209 | (-0.003, 0.001) |
| Intubation | | 2.610 | 0.959 | 0.001 | (0.373, 1.545) |
| sEOS | | 3.337 | 1.205 | <0.001 | (0.615, 1.795) |
| PDA | | 2.674 | 0.984 | 0.001 | (0.421, 1.546) |
| BUN>8.18 mmol/L | | 2.515 | 0.922 | 0.001 | (0.366, 1.478) |

^1^, comparison was made between no BPD and (mild, moderate and severe) BPD; ^2^, comparison was made between (no and mild) BPD and (moderate and severe) BPD; ^3^, comparison was made between (no, mild and moderate) BPD and severe BPD. Gr, gram; ALP, alkaline phosphatase; sEOS, (Suspected) early-onset sepsis; PDA, patent ductus arteriosus; BUN, blood urea nitrogen.
